# Histological Analysis of Sticky Tooth and Sticky Bone

**DOI:** 10.3390/jfb16070233

**Published:** 2025-06-25

**Authors:** Robert Dłucik, Marcel Firlej, Katarzyna Bogus, Daniel Dłucik, Bogusława Orzechowska-Wylęgała

**Affiliations:** 1 Dłucik Dental Clinic, 40-737 Katowice, Poland; dlucik@dlucik.pl; 2 Department of Orthodontics and Craniofacial Anomalies, Poznan University of Medical Sciences, 61-701 Poznan, Poland; marcel-firlej@wp.pl; 3 Department of Histology and Embryology, Medical University of Silesia, 40-752 Katowice, Poland; kbogus@sum.edu.pl; 4 Children’s Maxillofacial Surgery Clinic, Chair of Pediatric Surgery, Medical University of Silesia, 40-752 Katowice, Poland; boguslawa.wylegala@gmail.com

**Keywords:** sticky bone, sticky tooth, demineralized dentin matrix, mineralized dentin matrix, autogenous tooth graft, tooth grinders, equine bone, xenograft, allograft, bone augmentation materials, histological examination

## Abstract

Objective: This study aimed to compare the efficacy of Sticky Tooth (ST) derived from ground teeth and Sticky Bone (SB) based on equine bone and human allograft in maxillary bone defect regeneration through histological examination. Materials and Methods: Forty patients underwent maxillary alveolar ridge regeneration using four different biomaterials: Sticky Tooth processed with the BonMaker device (*n* = 10), Sticky Tooth prepared with the Smart Dentin Grinder (*n* = 10) Sticky Bone derived from an equine xenograft (*n* = 10), and Sticky Bone derived from human allografts (*n* = 10). CBCT imaging was performed preoperatively, post-regeneration, and during follow-up. Histological and quantitative statistical evaluation was conducted on biopsy samples obtained four months post-regeneration at the time of implant placement. Results: Successful alveolar ridge regeneration was achieved in all 40 patients. Histological analyses confirmed good integration between the biomaterials and bone tissue without signs of inflammation. Conclusion: Histological comparisons demonstrated that both ST and SB are effective biomaterials for bone regeneration. However, ST exhibited a faster bone healing process compared to xenograft and allograft SB.

## 1. Introduction

The key to successful bone regeneration is selecting the optimal biomaterial for implant site reconstruction. Although various bone grafting materials are available, autogenous bone remains the gold standard. However, its limited availability and the potential complications associated with harvesting procedures necessitate alternative solutions [1,2].

The use of mineralized dentin matrix (MDM) and demineralized dentin matrix (DDM) in bone defect treatment has been extensively studied, demonstrating high clinical effectiveness [3,4,5,6,7,8]. Autogenous dentin matrices possess both osteoconductive and osteoinductive properties due to their bone morphogenetic protein (BMP) content, distinguishing them from allografts, synthetic alloplasts, and xenografts [9,10,11,12,13,14]. Structurally, dentin has a chemical composition similar to bone [15]. DDM and MDM are sterile materials that can be processed and applied during a single patient appointment, with a processing time of less than 30 min [16]. The clinical use of ground, chemically processed, and demineralized teeth dates back to 2002 when Professor Masaru Murata first introduced this processing technique for human application [17]. A notable advantage of using autogenous teeth as a biomaterial is their cost-effectiveness as processing requires minimal investment.

In cases where autogenous tooth grafting is not possible (e.g., patients lacking extractable teeth), xenografts are used as the most common alternative [18]. These heterologous bone substitutes resemble human bone in chemical composition and morphology [19]. Bone consists of an inorganic component (approximately 70% hydroxyapatite crystals) and an organic component (primarily type I collagen) [20]. Bovine bone collagen shares ~95.9% homology with human bone collagen, while equine bone collagen exhibits ~95.2% similarity [21]. Xenografts demonstrate good mechanical properties and undergo remodeling and osteoconductivity, and some authors report their osteoinductive potential depending on the deantigenization process [22]. Clinical use of animal-derived biomaterials is well-documented in the literature [23,24,25,26,27]. While widely available, xenografts increase procedural costs and often require barrier membranes to maintain graft integrity and prevent soft tissue ingrowth [28].

Among the various grafting options available, allogeneic bone has emerged as a widely utilized material due to its inherent osteoconductive and osteoinductive properties. Sourced from genetically distinct donors of the same species, allogeneic grafts offer a biologically active scaffold that can support new bone formation without the need for harvesting autologous tissue [29]. Fresh or cryopreserved allografts are generally associated with enhanced mechanical strength and a greater capacity to promote osteogenesis. Due to the supercritical CO_2_-based treatment used by some allograft producers, the procedure is proven to be safe and the biomaterial can be stored in room temperature in a box for a few years. The processing technique, which utilizes supercritical CO_2_ for bone delipidation, combined with chemical oxidation removes residual proteins from the porous cancellous structure [30,31].

Platelet-rich fibrin (PRF) is increasingly used in dental implantology in different forms and preparation protocols [32]. PRF is a second-generation autologous blood-derived product containing multiple growth factors, including Platelet-Derived Growth Factors (PDGFs), Transforming Growth Factor-β (TGF-β), Bone Morphogenic Proteins (BMPs), and Vascular Endothelial Growth Factors (VEGFs) [33]. These factors play a crucial role in natural tissue healing. PRF is obtained by centrifuging the patient’s blood, and it can be compressed into membranes rich in growth factors. Studies indicate that PRF accelerates soft tissue healing after surgery, reduces postoperative swelling, enhances angiogenesis and cellular chemotaxis [34].

Sticky Bone (SB) is a combination of platelet-rich fibrin and a bone substitute, widely adopted in regenerative techniques [35,36]. A more recent innovation involves combining PRF with ground teeth, resulting in Sticky Tooth (ST) [37]. ST demonstrates excellent clinical outcomes, offering high material plasticity while maintaining form. Additionally, it provides osteoinductive and osteoconductive properties and accelerates healing due to its growth factor content. The present study aimed to conduct a histological analysis of dentin-derived grafts combined with PRF, processed using the BonMaker (BM—Korea Dental Solution, Busan, Republic of Korea), Smart Dentin Grinder (SDG—KometaBio, NJ, USA) equine-derived SB (Biogen mix—Bioteck, Arcugnano, Vincenza, Italy), and SB allografts (Biobank, Paris, France).

## 2. Materials and Methods

### 2.1. Study Design and Population

**Figure 1:**
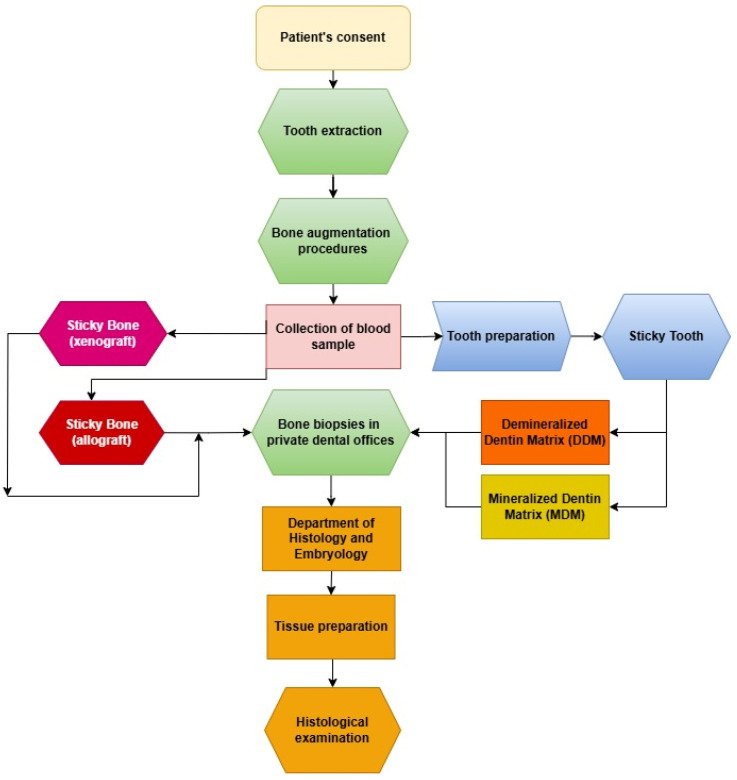
Flowchart illustrating study design.

**Figure 2:**
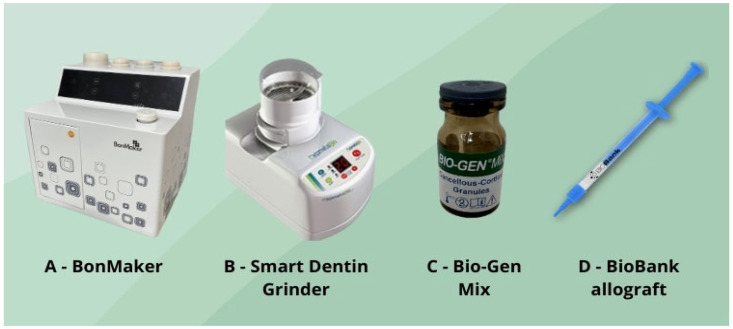
Tooth grinding machines: (**A**,**B**) equine bone Bio-Gen Mix (**C**) and BioBank allograft (**D**).

The study was conducted across three centers. The clinical component was performed in two private dental clinics, namely Dłucik Dental Clinic in Katowice, Poland, and Orto-Profil in Poznan, Poland, while histological analysis was conducted at the Department of Histology and Embryology of the Medical University of Silesia in Katowice. The study design was presented in the form of a flowchart (Figure 1). This study included 40 patients (28 females and 12 males) aged 24–83 years (mean age ± standard deviation: 50.75 ± 16.681 years). Patients were divided into four groups (*n* = 10 per group), each undergoing alveolar ridge augmentation using different biomaterials (Figure 2):

Group 1: Sticky Tooth-Demineralized Dentin Matrix (ST-DDM), processed using BM and Sticky Bone Selector(SBS-MEDIF, Warsaw, Poland);

Group 2: Sticky Tooth-Mineralized Dentin Matrix (ST-MDM), processed using SDG and SBS;

Group 3 (Control): SB from equine cancellous-cortical granules (Bio-Gen Mix);

Group 4 (Control): SB from human allograft cancellous-cortical granules (BioBank).

All patients underwent blood tests, including vitamin D3, calcium, cholesterol, creatinine, total cholesterol, and ALT levels, to qualify for the study. Bone density (D1, D2, D3, and D4) was assessed via CBCT (Table 1).

### 2.2. Inclusion and Exclusion Criteria

Inclusion Criteria:

- Age ≥ 18 years;
- Consent to participate and attend follow-ups (2 weeks, 4 weeks, and 3–4 months post-surgery);
- Good oral hygiene;
- Single tooth extraction without maxillary sinus communication;
- Clinical and radiological assessment confirming ridge defects.

Exclusion Criteria:

- Children;
- Systemic diseases;
- Poor oral hygiene;
- Odontogenic infections;
- Mental disorders;
- Smokers;
- Pregnant patients;
- Patients who refused to consent;
- Endodontically treated teeth;
- Oncology patients.

### 2.3. Preparation of Graft (In Vivo)

A total of 26 teeth were extracted, and in Groups 1 and 2, they were subjected to thorough decontamination on a specialized tooth-cleaning workstation. Initially, the extracted tooth was immersed for 3–5 min in a 3% hydrogen peroxide solution for preliminary disinfection. Subsequently, using diamond burs on a high-speed Kavo turbine, the tooth was cleaned of soft tissue, including the periodontal ligament, calculus, fillings, and any foreign bodies, in order to minimize variability and ensure material sterility. The cleaning procedure was conducted with Seliga surgical loupes at a 2.5× magnification. After the cleaning procedure, the extracted tooth was again placed in a 3% hydrogen peroxide solution for approximately 3 min and then thoroughly dried. For Group 1, the extracted teeth were prepared according to the manufacturer’s instructions for BM, which involved grinding the tooth in a sterile grinder, sieving (producing granules in the 500–1000 µm range), and placing the granules into a machine for demineralization with 3.5-5% hydrochloric acid (HCl), 70% ethanol, and 5% hydrogen peroxide for 20 min and 50 s. In Group 2, the tooth was placed in a sterile SDG grinder, ground (producing granules within the 300–1300 µm range), and then immersed in a sodium hydroxide solution with 20% ethanol for 10 min, followed by neutralization with phosphate-buffered saline. The total material preparation time was approximately 20 min. In the control group, commercially prepared granules of equine bone and allograft were used.

### 2.4. Surgical Procedure (In Vivo)

**Figure 3:**
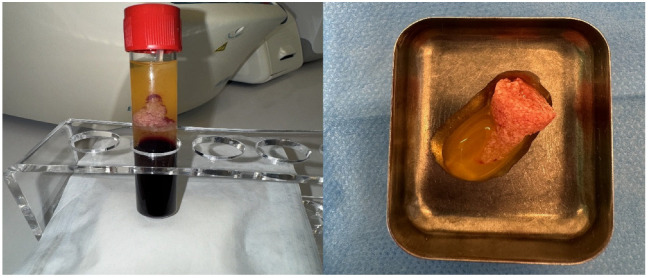
Clinical images demonstrating SB material.

**Figure 4:**
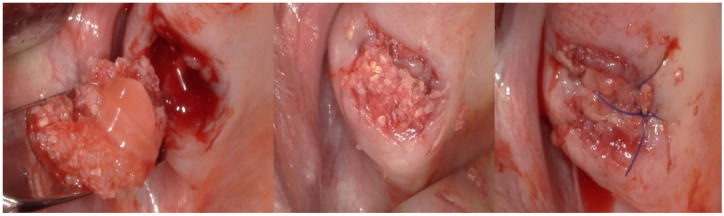
Panel of clinical photographs showing, in sequence, post-extraction socket, socket augmentation using ST, and wound after suturing.

**Figure 5:**
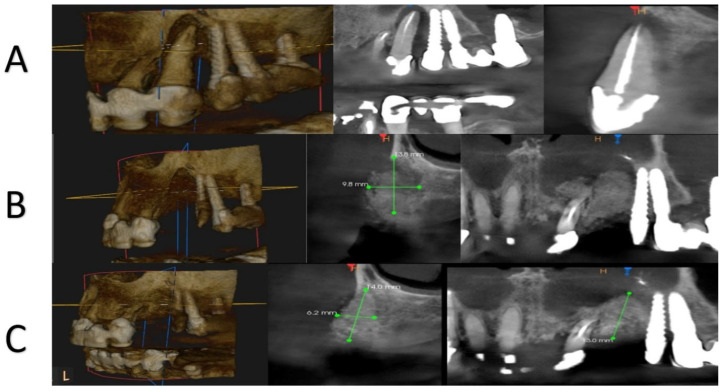
CBCT panel showing (**A**)—preoperative planning of extraction; (**B**)—immediate post-operative situation after socket filling with SB; (**C**)—condition four months after regeneration using SB.

For each patient, peripheral venous blood was collected into sterile tubes immediately prior to the surgical procedure. A Sticky Bone selector was inserted into each tube, followed by the addition of the respective graft material specific to the study group. The prepared tubes were then placed in a Win Union + centrifuge (Poldent, Warsaw, Poland) and centrifuged at 1300 rpm for 8 min. This protocol allowed for the formation of a fibrin-rich matrix embedding the graft particles, consistent with clinically accepted methods for PRF-based composite graft preparation. After obtaining the centrifuged preparations (Figure 3), the surgical procedure was initiated. All procedures were performed under antibiotic coverage with 1 g of amoxicillin combined with clavulanic acid and premedication consisting of one sachet of Nimesil (100 mg) 30–60 min before the surgery. Prior to the procedure, all patients rinsed their oral cavity with a chlorhexidine solution for 30 s. Local anesthesia was administered using articaine hydrochloride with the addition of norepinephrine at a concentration of 1:100,000. Subsequently, bone defects were filled with dentin particles and gently compressed (Figure 4). In Groups 1 and 2, the regenerated area was covered with PRF membranes, while in Groups 3 and 4, a Bioteck collagen membrane was used according to the manufacturer’s recommendations. The sites were closed for 14 weeks using non-absorbable, monofilament polyamide 5.0 surgical sutures. Cone-beam computed tomography (CBCT) examinations were conducted for all patients preoperatively, immediately postoperatively, and at a 4-month follow-up to assess treatment outcomes (Figure 5).

### 2.5. Bone Biopsies (In Vivo)

**Figure 6:**
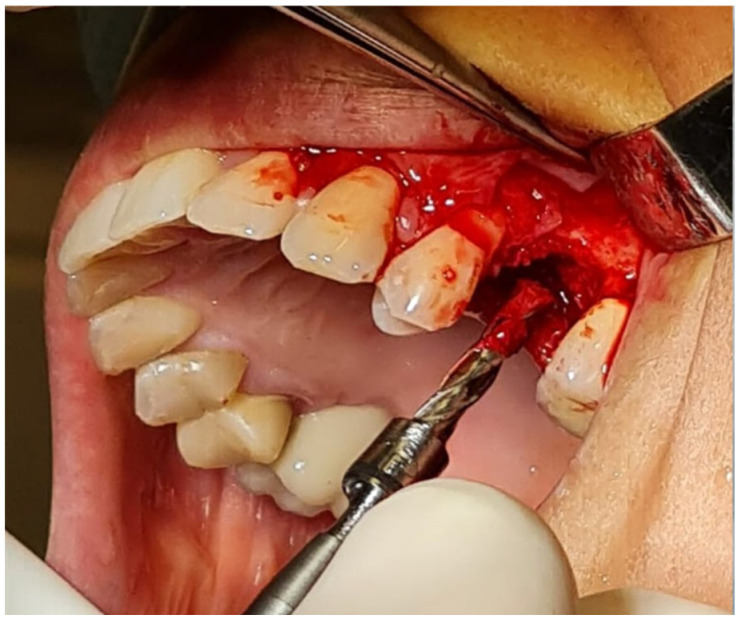
Example of bone block collection for histological examination.

Bone biopsies were performed on all 40 patients included in the study after a period of 4 months during the preparation of the implant bed using a Meisinger 4.0/5.0 trephine (Figure 6). An envelope incision was made along the gingival sulcus, and a mucoperiosteal full-thickness flap was raised to expose the alveolar ridge of the maxilla at the site of regeneration. The height of the bone blocks ranged from 8.5 to 10 mm. All biopsy specimens were placed in formaldehyde containers and subsequently sent to the Department of Histology and Embryology at the Medical University of Silesia in Katowice for an evaluation of the healing process.

### 2.6. Histological Procedure (In Vitro)

Upon receipt, the specimens were fixed in 10% neutral-buffered formaldehyde and decalcified for one week using TBD-1 Rapid Decalcifier, a solution containing EDTA and diluted hydrochloric acid. Following decalcification, standard histological processing was conducted. This involved graded ethanol dehydration (70–100%) via the Chandon Citadela 2000 automated system, paraffin embedding using the TEC-2800 Embedding Center, and microtome sectioning into 5 μm slices with the Microm HM 350S. The tissue sections were then mounted on glass slides, deparaffinized, rehydrated, and stained with either hematoxylin and eosin (H&E) or Masson’s trichrome. Imaging was carried out using an Olympus BX43ZE microscope (Evident, Hachioji, Japan) coupled with CellSense software (version 4.4).

### 2.7. Quantitative Histological Analysis

Statistical analysis was performed on all samples in the study using the data analysis software system Statistica (TIBCO Software Inc., 2017, version 13). Due to the lack of normal distribution (according to the Shapiro–Wilk test) and homogeneity of variance (according to Levene’s test), the mean differences between groups were analyzed using the Kruskal–Wallis test followed by Dunnett’s post hoc test.

## 3. Results

### 3.1. Histological Examination

#### 3.1.1. BonMaker ST 

**Figure 7:**
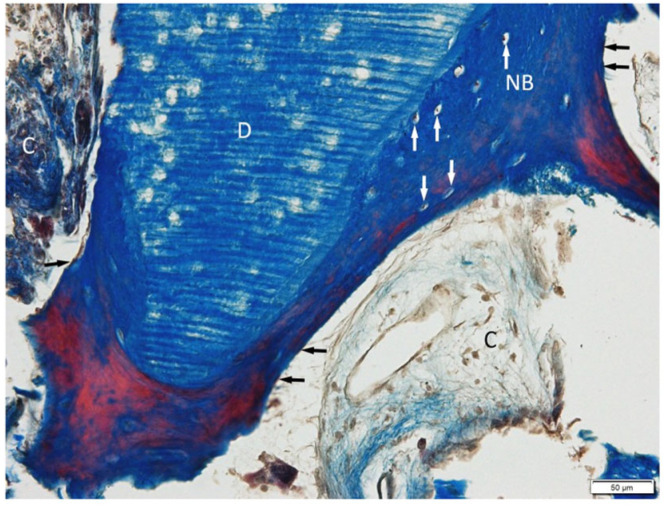
The dentin graft (D) is well connected with newly formed bone (NB). Osteocytes (white arrows) and osteoblasts (black arrows) are present. Soft connective tissue (C). Sample processed by BM, Masson Trichrome staining. Objective magnification ×20.

**Figure 8:**
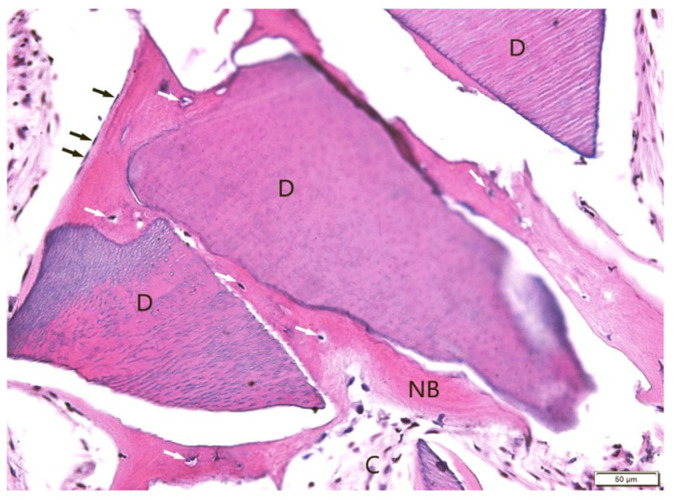
Sample stained with H&E (BM). Dentin graft (D) surrounded by and partially connected with newly formed bone (NB). White arrows: osteocytes, black arrows: osteoblasts. Soft connective tissue (C). Objective magnification ×20.

Histological evaluation using Masson’s trichrome (Figure 7) and H&E (Figure 8) staining revealed the successful incorporation of dentin graft particles (D) into the maxillary bone. Most particles were surrounded by newly formed bone (NB), while a smaller fraction remained embedded in vascularized connective tissue (C). Osteoblasts (black arrows) were observed on trabecular surfaces, and osteocytes (white arrows) were present within lacunae, indicative of active bone remodeling and maturation. Masson’s trichrome clearly differentiated non-mineralized collagen-rich areas (blue) from zones undergoing mineralization (red). No signs of inflammatory response or abnormal tissue morphology were detected.

#### 3.1.2. Smart Dentin Grinder ST 

**Figure 9:**
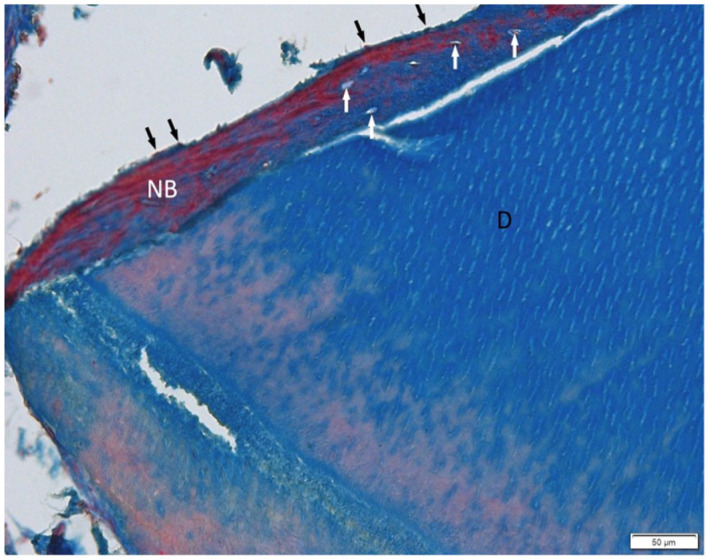
Sample processed by SDG, Masson Trichrome staining. The dentin graft (D) is surrounded by newly formed bone (NB). Osteocytes (white arrows) and osteoblasts (black arrows) are clearly presented here. Objective magnification ×20.

**Figure 10:**
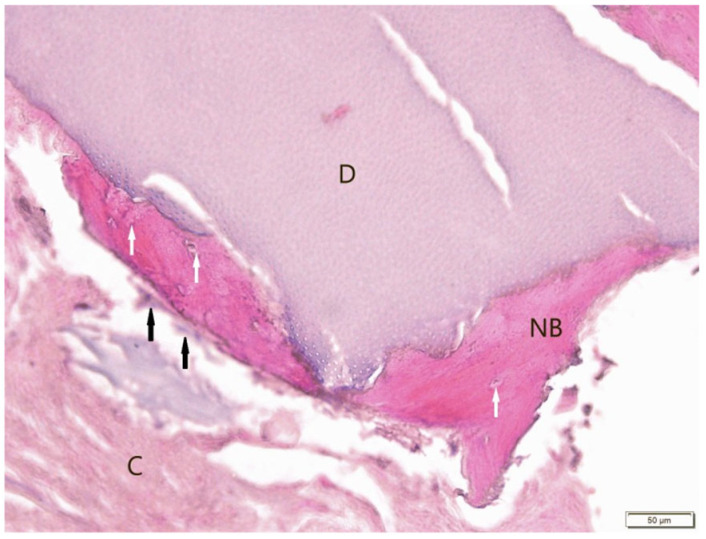
Tissue from a patient processed by SDG, stained with H&E. The dentin particle (D) is surrounded by newly formed bone (NB) and soft connective tissue (C). White arrows: osteocytes; black arrows: osteoblasts. Objective magnification ×20.

Sections stained with Masson’s trichrome (Figure 9) and H&E (Figure 10) showed that ground dentin fragments (D) were predominantly surrounded by newly deposited bone matrix (NB), with limited involvement of connective tissue (C). The interface between dentin and adjacent bone was well defined. Masson’s trichrome demonstrated direct apposition of collagen-rich bone matrix (blue) to dentin particles. Typical bone-forming cells—osteoblasts and osteocytes—were clearly visualized (Figure 9 and Figure 10).The absence of an inflammatory response further supports the favorable biocompatibility and osteoconductive properties of the material.

#### 3.1.3. Bio-Gen Mix Xenograft SB 

**Figure 11:**
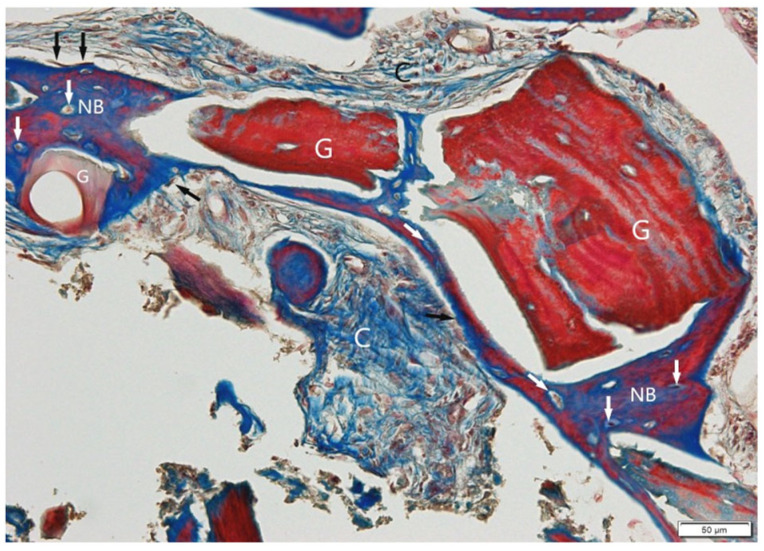
Bio-Gen Mix sample, Masson Trichrome staining. A perfect connective between the granules (G) and newly formed bone (NB) is visible here (black arrows: osteoblasts; white arrows: osteocytes). Mineralized bone (MB), soft connective tissue (C). Objective magnification ×20.

**Figure 12:**
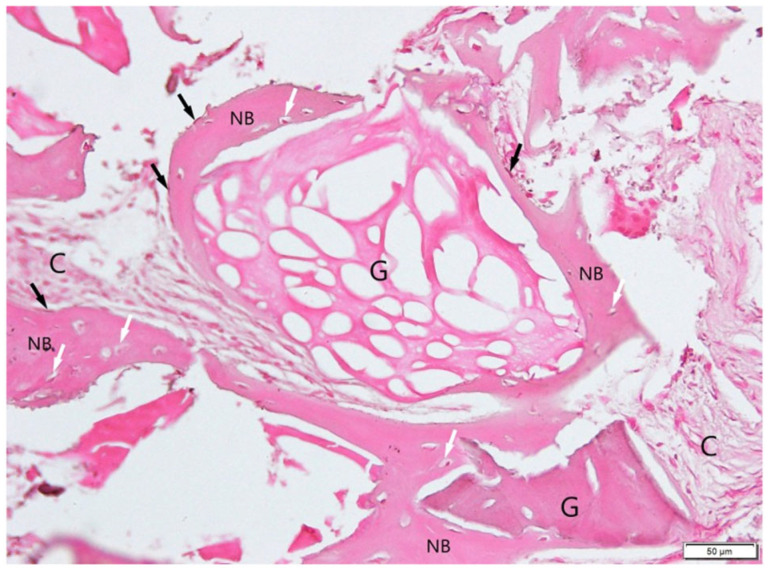
Tissue from a patient augmented by Bio-Gen Mix and stained with H&E. A good connection between the granules (G) and newly formed bone (NB) is observed. At this magnification, remaining granules of the graft in the soft tissue (C) are clearly visible. White arrows: osteocytes; black arrows: osteoblasts. Objective magnification ×20.

Granules of Bio-Gen Mix (G) were mostly incorporated into new bone, although some remained within soft connective tissue (C). Masson’s trichrome (Figure 11) demonstrated a clear distinction between collagen-rich (blue) and mineralizing regions (red), presenting the boundary between the xenograft and new bone. H&E (Figure 12) and trichrome staining revealed active osteogenesis, with osteoblasts (black arrows) lining the bone surfaces and osteocytes (white arrows) embedded in the matrix. There was no histological evidence of inflammation or adverse tissue reaction.

#### 3.1.4. BioBank Allograft SB

**Figure 13:**
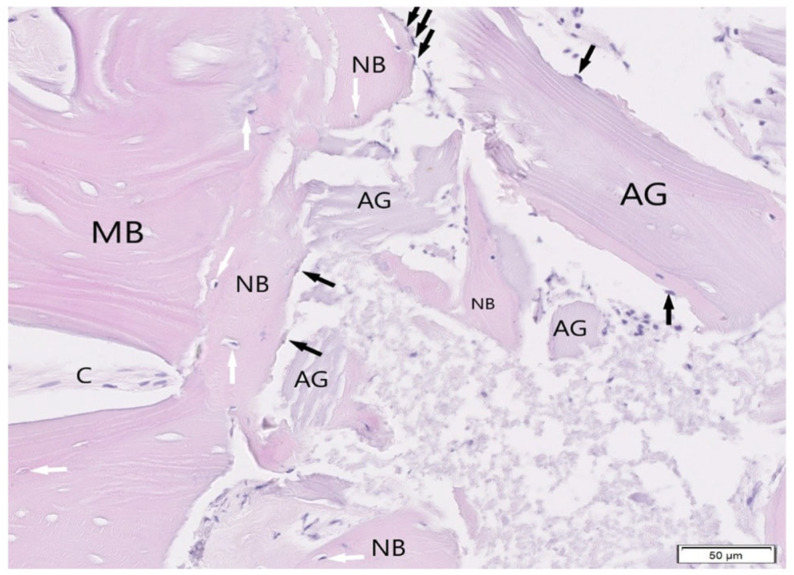
The allografts (AG) partially surrounded by newly formed bone (NB). Osteoblasts (black arrows) and osteocytes (white arrows) are clearly visible at bone tissue. Single allograft in the connective tissue (C). Mineralized bone trabeculae (MB). Tissue stained with H&E, objective magnification ×20.

**Figure 14:**
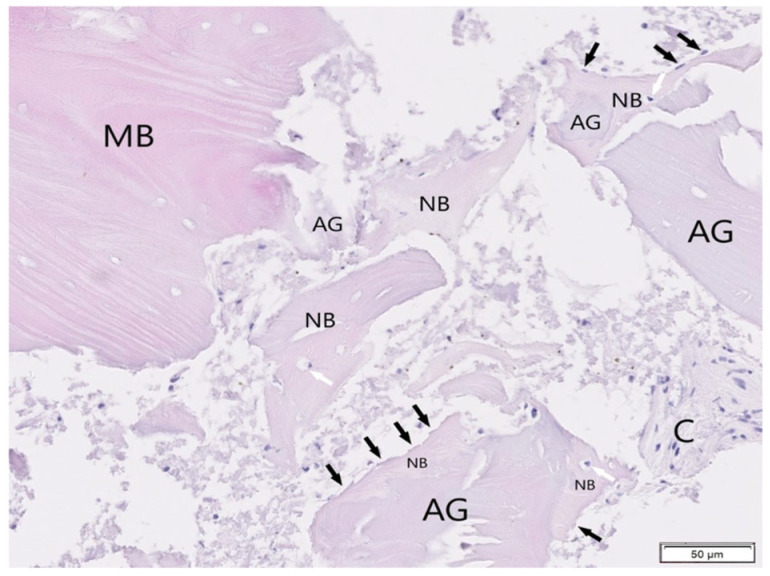
Tissue from patient where allografts were used (AG). Most fragments are surrounded by and connected with newly formed bone (NB, osteoblasts-black arrows, osteocytes-white arrows are presented). Connective tissue (C), mineralized bone trabeculae (MB). H&E staining, objective magnification ×20.

The BioBank allograft samples (H&E staining) exhibited trabecular bone with normal lamellar architecture and homogeneous mineralization (Figure 13). Osteoblast rimming was present along bone margins, indicating ongoing bone formation. Most graft fragments were surrounded by new bone containing osteocytes in lacunae, while some portions remained in non-inflamed connective tissue (Figure 14). No pathological changes were observed, suggesting successful integration and tissue compatibility.

### 3.2. Histological Evaluation of Graft Integration with Bone Tissue

Quantitative histological analysis was performed to assess the degree of integration of graft materials with newly formed bone tissue in the samples. The results were expressed as the percentage of graft material surrounded by bone versus the portion remaining within connective tissue (Table 2). In the ST-DDM (demineralized dentin matrix) group, 75.86% of the graft material was embedded in bone, while 24.14% remained unintegrated within connective tissue. In the ST-MDM (mineralized dentin matrix) group, 69.10% of the material was surrounded by bone, with 30.90% not incorporated. The SB xenograft group showed 56.25% bone-embedded material and 43.75% unintegrated graft. Similarly, in the SB allograft group, 58.30% of the graft was embedded in bone, whereas 41.70% remained in connective tissue. These findings indicate significant differences among the evaluated materials, which may influence their clinical applicability in bone regeneration procedures.

### 3.3. Evaluation of Healing Process

During follow-up visits, 40 patients (all from groups 1, 2, and 4 and 8 patients from group 3) showed good healing of soft tissues with no swelling observed. Two patients from group 3 presented graft exposure due to wound edge separation. These patients underwent ozone therapy, laser therapy (Smart M ST diode laser, Lasotronix, Piaseczno, Poland), regular mouth rinsing with chlorhexidine solution, and the application of ointment (Cicalium, Pierre Fabre Oral Care, Lavaur, France) until secondary wound healing occurred. None of the 40 patients reported significant pain after procedures involving ST and SB. Four months following the regenerative procedure, successful implantation was achieved in all 40 patients. In three patients from the SB xenograft group, the bone appeared less mineralized (softer) with visible granules of non-resorbed equine bone compared to the patients treated with ST and the SB allograft.

## 4. Discussion

Bone loss associated with missing teeth is an inevitable process that can be reversed through the application of various biomaterials [38]. The role of SB in reconstructive surgery procedures has been recognized by many clinicians. A literature review published in 2024, which reviewed the work of 12 authors, demonstrated the positive effects of using SB in regenerative procedures [36]. The researchers reported that the use of this technique increases bone volume (both vertically and horizontally), accelerates healing (of both hard and soft tissues), reduces bone resorption during the healing period, improves procedure predictability, and results in a quicker transition to implantation. In another study, based on histological findings, researchers showed that 6 months after the application of SB in the maxilla (deproteinized bovine bone material), significant remodeling of the material occurred, and the technique successfully restored the proper dimensions of the alveolar ridge prior to implant placement [39].

Not all patients may accept the use of xenografts or allogeneic materials, making the use of autogenous tooth grafts in the form of ST a promising alternative. Combining autologous dentin grafts with platelet-rich fibrin (PRF) creates a “Sticky Tooth” composite, improving graft stability and clot formation. A 2022 histological study involving seven patients demonstrated the use of ST processed via SDG. Tooth-derived granules demonstrate osteoinductive properties due to retained dentin matrix proteins like BMP-2, enhancing bone regeneration [40]. Dentin’s microarchitecture provides osteoconductive scaffolding, with particle size optimizing cell migration and vascular invasion. Histological analysis revealed greater new bone formation in sockets treated with Sticky Tooth versus control groups with bovine xenografts. After 6 months of regeneration, it was shown that all patients had developed new trabecular bone, making this a viable alternative to traditional grafting materials [40].

In a study of 32 patients with 16 in each group, researchers compared dentin graft vs. untreated extraction sockets. After four months, the study group exhibited significantly less dimensional change and better tissue remodeling compared to the control, as confirmed by CBCT and histological analysis. Autogenous dentin grafts reduce alveolar ridge resorption compared to untreated sockets, preserving dimensions for implant placement [13]. Other authors also use patients’ dentin prior extraction to allow for implant site preparation through the tooth with compaction autografting of bone and dentin with osseodensification burs, thereby improving implant primary stability and its subsequent early healing [41,42].

Dentin is a complex, porous tissue composed primarily of hydroxyapatite (70%), with smaller proportions of organic material (20%) and water (10%). Treatment with agents like EDTA, hydrochloric acid, or nitric acid selectively removes mineral content while preserving collagen and bioactive proteins, forming an osteoinductive matrix. Demineralization increases dentin’s porosity, accelerating the release of calcium/phosphate ions to stimulate osteoblast activity. As a result, demineralized dentin may serve as a promising natural scaffold for promoting the regeneration of dental and alveolar bone tissues [43]. However, excessive demineralization can compromise the mechanical integrity of the graft [44]. Also, the lack of standardized demineralization methods can lead to inconsistent graft quality [45]. In summary, while DDM offers enhanced biological activity conducive to bone regeneration, it may lack the mechanical strength of non-demineralized grafts. Conversely, non-demineralized dentin provides structural support but may be less effective in stimulating new bone formation. The intact mineral matrix may hinder the release of growth factors, reducing the graft’s ability to stimulate new bone formation [46]. The choice between these materials should be guided by the specific clinical requirements of the defect site. PRF studies validated fibrin’s role in accelerating osteogenesis over time [47,48]. Extensive research has highlighted the benefits of PRF in periodontal and soft tissue healing; however, high-quality studies providing clear evidence of its effectiveness in hard tissue bone regeneration are still limited [49]. Combining autologous dentin grafts with platelet-rich fibrin (PRF) creates a “Sticky Tooth” composite, improving graft stability and clot formation [40].

In this study, we aimed to demonstrate the differences between ST-DDM (group 1), ST-MDM (group 2), and SB (groups 3 and 4) in histological images 4 months after the alveolar ridge regeneration procedure, which indirectly addressed the issue of patients needing to achieve final prosthetic work in the shortest possible time. When comparing ST-DDM with ST-MDM, no differences were observed in the histological images. When comparing ST and SB, we observed that SB preparations contained more non-remodeled granules surrounded by connective tissue compared to ST preparations. This suggests that ST undergoes faster calcification. It should be noted that the standard healing time for equine bone and human allografts in the maxilla is approximately 6 months; however, implant placement was successfully performed in all patients from the SB group after 4 months. The presence of osteogenic cells (osteoblasts and osteocytes) and good integration of bone with the tested materials (ST and SB) demonstrates the effectiveness of the described techniques. No pathological changes were observed in any of the biopsy preparations, indicating that both techniques are promising and safe for clinical use. The results of our study align with the findings of other authors, although the literature on ST remains limited. A limitation of this study is that bone biopsies were conducted on all participants only after a four-month period. Additional studies with greater numbers of samples and patients are needed to assess how the grafted areas undergo resorption and regeneration over a longer time.

Other study limitations were the lack of histomorphometric analysis (the inability to obtain histologically comparable samples), biomechanical testing, and periodontal ligament stem cell (PDLSC) evaluation. PDLSCs represent a promising cellular resource for bone regeneration, especially in the treatment of periodontal diseases and applications in dental tissue engineering. These cells are easily obtainable and possess significant osteogenic differentiation potential. As such, PDLSCs hold considerable therapeutic value for the reconstruction of alveolar bone and the regeneration of periodontal structures [50]. The grinding process (300–1300 µm particle size) and chemical treatments (HCl/NaOH) were optimized to preserve dentin’s collagen and BMP content while intentionally denaturing periodontal stem cells as prior studies show limited viability post-mechanical processing. In the current protocol, the teeth used in the Sticky Tooth preparation were thoroughly cleaned and processed using standard protocols to remove any remaining soft tissues, including the periodontal ligament, in order to minimize variability and ensure material sterility. While stem cell preservation could enhance osteogenesis, our protocol prioritizes bacterial decontamination and graft stability. Future studies could explore gentler processing for stem cell viability. In summary, the process of demineralization is crucial for activating biologically important substances within the graft. This study quantitatively evaluated the integration of various graft materials with newly formed bone tissue. The results show that the ST-DDM group had the highest bone integration (75.86%), followed by ST-MDM, the SB allograft, and the SB xenograft, indicating material-dependent differences that could impact their clinical use in bone regeneration. Partially demineralizing the graft can offer a beneficial compromise—maintaining vital proteins and growth factors while improving its ability to support new bone growth—making it a potential choice for smaller defects or specific clinical situations [9,51]. On the other hand, over-demineralization can lead to increased resorption of the graft as the exposed collagen becomes more vulnerable to enzymatic degradation. Non-demineralized grafts may be suitable when slower resorption is desired or in larger defects [52]. According to the literature the volume of the newly formed mineralized bone does not increase over time, even after 20 years of sinus grafting with xenografts, and remains at 22.53% at 12 months and at 22.05 after 20 years [53]. DDM’s osteoinductivity likely stems from BMP-2 release during demineralization, whereas xenografts rely on collagenous osteoconduction. Equine xenografts may be less effective in bone augmentations when compared to dentin grafts or allografts.

## 5. Conclusions

Among the various biomaterials available for bone regeneration, selecting the appropriate one can be challenging. The use of equine bone and allografts in the form of Sticky Bone (SB), along with ground dentin matrix in the form of Sticky Tooth (ST), enables effective and rapid regeneration of bone defects. ST-DDM, ST-MDM, and SB provide comparable results in clinical application in short-term follow-ups. Demineralized dentin and allografts may have comparable results in volume stability, and mineralized dentin seems to be more effective in larger defects due to longer osteoconductivity and longer resorption time. Equine xenografts may also provide longer osteoconductivity, but its transformation ability into a new bone in the long term is questionable. A histological evaluation of 40 bone biopsies demonstrated that ST grafts achieved greater new bone formation than SB xenografts, while SB allografts achieved better new bone formation than SB xenografts, fulfilling the study’s aim to compare biomaterial efficacy (Table 2). The use of ST is significantly less expensive than purchasing SB. Further studies on ST and SB, with an emphasis on histomorphometric analysis, should be conducted to obtain more data in this area. This study did not assess mechanical properties or provide longitudinal data beyond 4 months; therefore, further evaluation is required in long-term observations.

## Figures and Tables

**Table 1:** The mean blood parameters of patients across all groups; the asterisk (*) indicates the average.

Graph 3.	Type of Biomaterial	Vitamin D3 Level *	Calcium Level *	Creatinine Level *	Total Cholesterol *	ALT Level *	Bone Density *
1	ST-DDM	46.3 ng/mL	9.15 mg/dL	0.90 mg/dL	189 mg/dL	21 U/L	D2
2	ST-MDM	45.2 ng/mL	8.94 mg/dL	0.98 mg/dL	196 mg/dL	19 U/L	D2
3	SB Xenograft	40.1 ng/mL	10.3 mg/dL	1.02 mg/dL	185 mg/dL	18 U/L	D2
4	SB Allograft	42.8 ng/mL	9.40 mg/dL	0.94 mg/dL	191 mg/dL	22 U/L	D2

**Table 2:** The results of the quantitative histological analysis.

Group	Percentage of Graft Material Embedded in Bone	Percentage of Graft Material Unintegrated within Connective Tissue
C1 (SB Xenograft)	56.25%	43.75%
C2 (SB Allograft)	58.30%	41.70%
ST-DDM	75.86%	24.14% ^1^
ST-MDM	69.10% ^2,3^	30.90% ^4^

^1^ Differences were considered statistically significant at *p* < 0.05 (relative to control C1 group). ^2^ Differences were considered statistically significant at *p* < 0.0001 (relative to control C1 group). ^3^ Differences were considered statistically significant at *p* < 0.0001 (relative to control C2 group). ^4^ Differences were considered statistically significant at *p* < 0.05 (relative to control C2 group).

## Data Availability

The original contributions presented in this study are included in the article; further inquiries can be directed to the corresponding author.
